# Supplementary material for: The causal role of circulating inflammatory markers in osteoporosis: a bidirectional Mendelian randomized study
Source: Front Immunol. 2024 Jul 18;15:1412298. doi: 10.3389/fimmu.2024.1412298 (PMC11291241; doi:10.3389/fimmu.2024.1412298)
Supplement: Supplementary file 1 [file DataSheet_1.zip › supplementary material/CIM-OSTEOPOROSIS /GCST90274803.csv_forest.pdf]

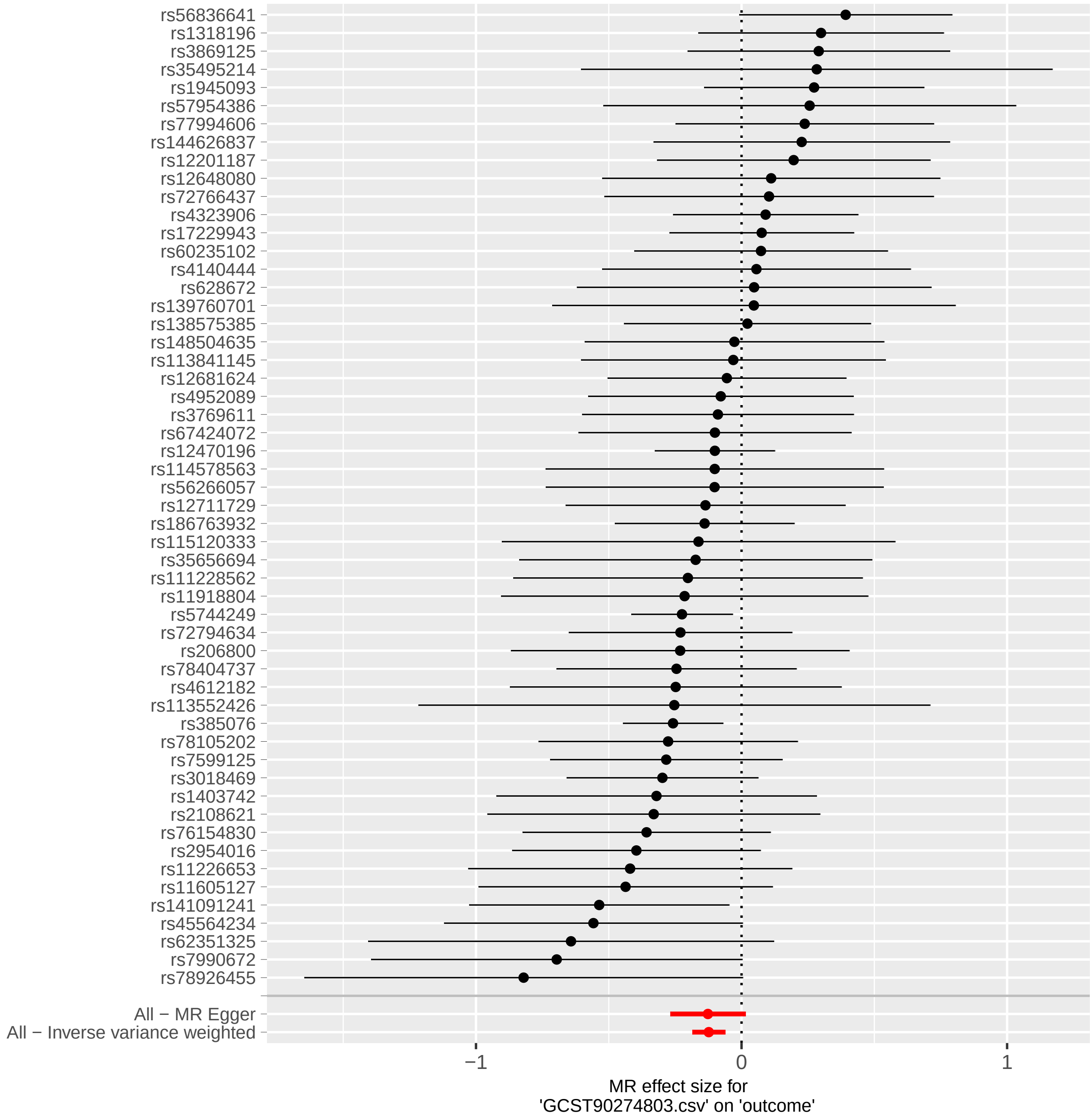
rs56836641
rs1318196
rs3869125
rs35495214
rs1945093
rs57954386
rs77994606
rs144626837
rs12201187
rs12648080
rs72766437
rs4323906
rs17229943
rs60235102
rs4140444
rs628672
rs139760701
rs138575385
rs148504635
rs113841145
rs12681624
rs4952089
rs3769611
rs67424072
rs12470196
rs114578563
rs56266057
rs12711729
rs186763932
rs115120333
rs35656694
rs111228562
rs11918804
rs5744249
rs72794634
rs206800
rs78404737
rs4612182
rs113552426
rs385076
rs78105202
rs7599125
rs3018469
rs1403742
rs2108621
rs76154830
rs2954016
rs11226653
rs11605127
rs141091241
rs45564234
rs62351325
rs7990672
rs78926455
All − MR Egger
All − Inverse variance weighted
−1
0
1
MR effect size for
'GCST90274803.csv' on 'outcome'
